# Development of AAVLP(HPV16/31L2) Particles as Broadly Protective HPV Vaccine Candidate

**DOI:** 10.1371/journal.pone.0039741

**Published:** 2012-06-27

**Authors:** Karen Nieto, Margit Weghofer, Peter Sehr, Mirko Ritter, Sebastian Sedlmeier, Balasubramanyam Karanam, Hanna Seitz, Martin Müller, Markus Kellner, Markus Hörer, Uwe Michaelis, Richard B. S. Roden, Lutz Gissmann, Jürgen A. Kleinschmidt

**Affiliations:** 1 Research Program Infection and Cancer, German Cancer Research Center, Heidelberg, Germany; 2 MediGene AG, Planegg/Martinsried, Germany; 3 Chemical Biology Core Facility, European Molecular Biology Laboratory, Heidelberg, Germany; 4 Department of Pathology, Johns Hopkins University, Baltimore, Maryland, United States of America; International Centre for Genetic Engineering and Biotechnology, Italy

## Abstract

The human papillomavirus (HPV) minor capsid protein L2 is a promising candidate for a broadly protective HPV vaccine yet the titers obtained in most experimental systems are rather low. Here we examine the potential of empty AAV2 particles (AAVLPs), assembled from VP3 alone, for display of L2 epitopes to enhance their immunogenicity. Insertion of a neutralizing epitope (amino acids 17–36) from L2 of HPV16 and HPV31 into VP3 at positions 587 and 453, respectively, permitted assembly into empty AAV particles (AAVLP(HPV16/31L2)). Intramuscularly vaccination of mice and rabbits with AAVLP(HPV16/31L2)s in montanide adjuvant, induced high titers of HPV16 L2 antibodies as measured by ELISA. Sera obtained from animals vaccinated with the AAVLP(HPV16/31L2)s neutralized infections with several HPV types in a pseudovirion infection assay. Lyophilized AAVLP(HPV16/31L2) particles retained their immunogenicity upon reconstitution. Interestingly, vaccination of animals that were pre-immunized with AAV2 - simulating the high prevalence of AAV2 antibodies in the population - even increased cross neutralization against HPV31, 45 and 58 types. Finally, passive transfer of rabbit antisera directed against AAVLP(HPV16/31L2)s protected naïve mice from vaginal challenge with HPV16 pseudovirions. In conclusion, AAVLP(HPV16/31L2) particles have the potential as a broadly protective vaccine candidate regardless of prior exposure to AAV.

## Introduction

Persistent high-risk human papillomavirus (HPV) infection has been linked to the development of cervical cancer [1]. Attempts to prevent infection with the most prevalent high risk HPV types – HPV16 and HPV18 – led to the development of two highly efficacious HPV vaccines that comprise virus-like particles (VLPs) assembled from the major capsid protein L1. However these vaccines target only two of the 15 high-risk HPV types, responsible for 70–80% of cervical cancer cases. The prevention of 96% of cervical cancer would require the immunity against at least 7 high-risk HPV types (HPV16, 18, 31, 33, 45, 52 and 58), but this increases the cost and complexity of manufacture [2]. Future vaccines should therefore attempt to extend immunization against other high-risk HPV types. For broad application also in developing countries they should be provided inexpensively in a temperature stable formulation.

The minor capsid protein L2 harbors several regions that can be targeted by neutralizing antibodies [3]–[5]. One of which, located at amino acid position 17–36 comprises a major cross-neutralizing epitope and thus represents an attractive candidate antigen for broadly protective vaccination [6]–[8]. However, the major challenge in using the L2 protein as vaccine antigen is its rather poor immunogenicity compared to L1 VLP. It has been attempted to increase the immunogenicity of L2, by the use of different scaffolds [3], [6], [9], linking of L2 to TLR agonists [10] or by using a concatenated L2 N-terminal fragment [11].

Adeno-associated virus (AAV) belongs to the parvovirus family harboring a single-stranded DNA genome of approximately 4.7 kb. AAV particles that are very stable over a wide range of pH and temperature have a diameter of 25 nm, and are composed of 60 capsid protein subunits, designated VP1, VP2, and VP3 [12]. A recent study demonstrated that assembly of AAV capsids requires the expression of a virally encoded assembly-activating protein (AAP) [13]. Moreover, co-expression of VP3 with AAP allows the efficient production of virus-like particles (AAVLPs) composed of only the major capsid protein VP3 [13]. These capsids adopt an identical external capsid structure as VP1, VP2 and VP3 containing capsids [14] and represent a preferred scaffold for peptide vaccination, because of their simplicity and their inability to transfer genes which may inadvertently have been packaged in such AAVLPs. Due to the highly structured and repetitive presentation of epitopes on the capsid (60 times), combined with the intrinsic immunogenicity of AAV [15], potent B-cell responses against the peptide presented at immunogenic capsid positions can be expected. So far, AAV capsids have not been analyzed for this purpose.

Here we show for the first time the potential to present B-cell epitopes on AAVLP capsid surface. We generated AAVLPs displaying HPV16 L2 epitopes in position 587 and HPV31 L2 epitopes in position 453. Our results demonstrate cross-neutralizing responses against several HPV types and therefore suggest AAVLP(HPV16/31L2) particles as a broadly protective vaccine candidate.

## Results

### Production of AAVLP(HPV16/31L2) Particles

**Figure 1:**
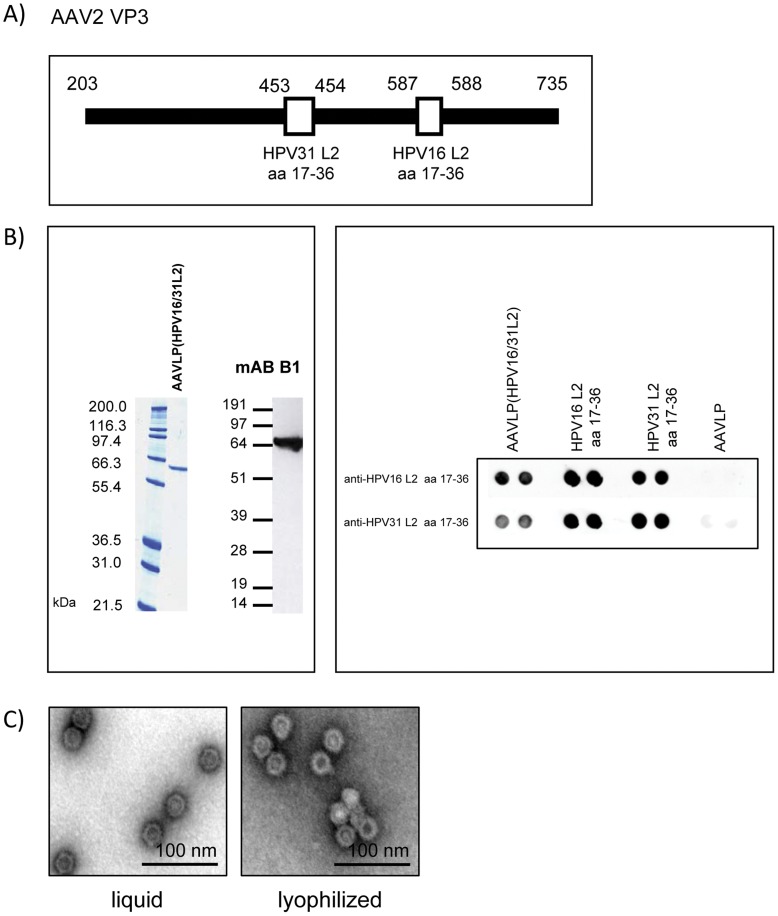
Generation and production of AAVLP(HPV16/31L2). A) Schematic presentation of the chimeric VP3 protein with the insertions of the HPV31 and HPV16 L2 peptides. The amino acid residues refer to the VP1 protein sequence. Solid lines indicate the VP3 protein and open bars the L2 peptides. B) Analysis of the purified AAVLP(HPV16/31L2) particles by SDS- PAGE, coomassie blue staining and Western blotting with the monoclonal antibody B1 directed against VP3. Native dot blots with polyclonal rabbit sera to the HPV16 and 31 L2 aa 17–36 peptides, AAVLP particles and the AAVLP(HPV16/31L2) particles. Dot blots are done in duplets. C) TEM of the purified particles.

Adeno-associated virus serotype 2 (AAV2) capsids tolerate insertion of peptides in their antigenic region at position 587 or 453 [16]–[22] that are attractive sites for presenting B-cell epitopes.The expression of only two AAV proteins, VP3 and AAP, allows the formation of VP3-VLPs (AAVLPs). These observations suggest that recombinant AAVLPs could be generated with insertions of peptides in positions 587 and 453. After immunization with HPV16 L2 peptide 17–36 a broad range of cross- neutralizing antibodies was generated, but low or no antibodies against HPV31 [23]. Since HPV L2 17–36 aa sequences of HPV16 and 31 are clearly different, we incorporated both HPV L2 peptides into AAVLP particles at position 587 and 453 (figure 1A). Stretches of 3 or 5 glycine residues were added to the N and C termini of the respective peptides for optimal surface display and several amino acids resulting from restriction enzyme sites used for cloning increased the size of inserted peptide sequences to 35 aa (position 453) and 31 aa (position 587). The amino acids at positions 585 and 588 were substituted. These R585A/R588A substitutions increased the titers of AAVLP particles with inserts. SDS-PAGE and Western blot analysis of AAVLPs confirmed the identity and size (approximately 66 kDa of AAVLP(HPV16/31L2) VP3) of the purified protein (figure 1B). Presentation of the inserted HPV L2 peptides on the AAVLP particles were demonstrated with L2-specific rabbit sera in a dot blot assay under native conditions. However, since these sera cross-react with the HPV16 and HPV31 displayed peptides, exposure of both of them could not be unambiguously proven. As shown in figure 1C, proteins assembled into VLPs of ∼25–30 nm in diameter. In conclusion, the insertions of 35 aa (position 453) and 31 aa (position 587) into the capsid protein did not interfere with VLP assembly.

### Immunogenicity of AAVLP(HPV16/31L2) Particles in C57BL/6 Mice

**Figure 2:**
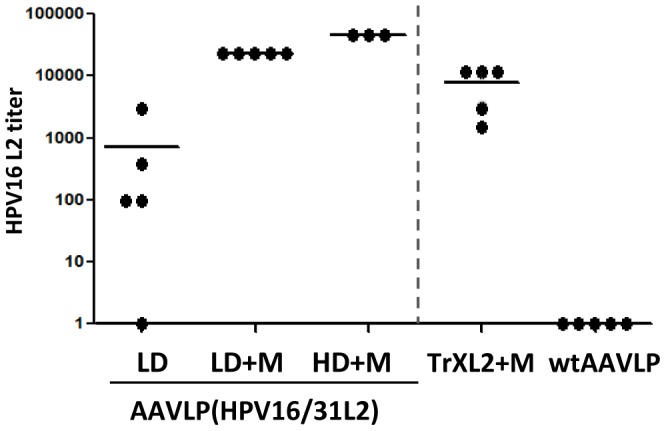
Detection of HPV16 L2-specific antibody titers in C57BL/6 mice vaccinated with AAVLP(HPV16/31L2). C57BL/6 mice (5 per group) vaccinated twice intramuscularly with AAVLP(HPV16/31L2) particles either with a low dose (LD) (1E+11 particles equivalent to 0.6 µg of protein) or high dose (HD) (5E+12 particles equivalent to 30 µg) with or without an adjuvant (+M) (montanide ISA 51). As positive control, mice were immunized three times subcutaneously with Thioredoxin-L2(20–38)_3_ (TrXL2) (50 µg). wtAAVLP particles served as negative control. HPV16 L2-specific antibodies were measured by GST-L2 ELISA six weeks after the first immunization. Data of individual mice are shown. Mean titers of each group are shown by horizontal bars. Data are expressed as the reciprocal titers of individual mice.

In order to analyse the HPV16 L2-specific immune response induced by AAVLP(HPV16/31L2) particles we immunized C57BL/6 mice either with a low dose (LD) (1E+11 particles equivalent to 0.6 µg of protein) or high dose (HD) (5E+12 particles equivalent to 30 µg) with or without adjuvant (+M) (montanide ISA 51). Thioredoxin-L2(20–38)_3_ (TrXL2) [3] (50 µg) was used as positive control, and wtAAVLP particles served as a negative control. Anti-HPV16 L2 antibodies were measured by GST-L2 ELISA six weeks after the first immunization. As shown in figure 2, mice immunized with LD+M or HD+M developed 25 and 50 fold higher HPV16 L2-specific antibodies titers, respectively, than mice immunized with a LD of particles without adjuvant. Interestingly, there was little difference between the mice immunized with LD+M and mice immunized with HD+M. Sera of all mice vaccinated with TrXL2+M had HPV16 L2-specific antibodies.

**Figure 3:**
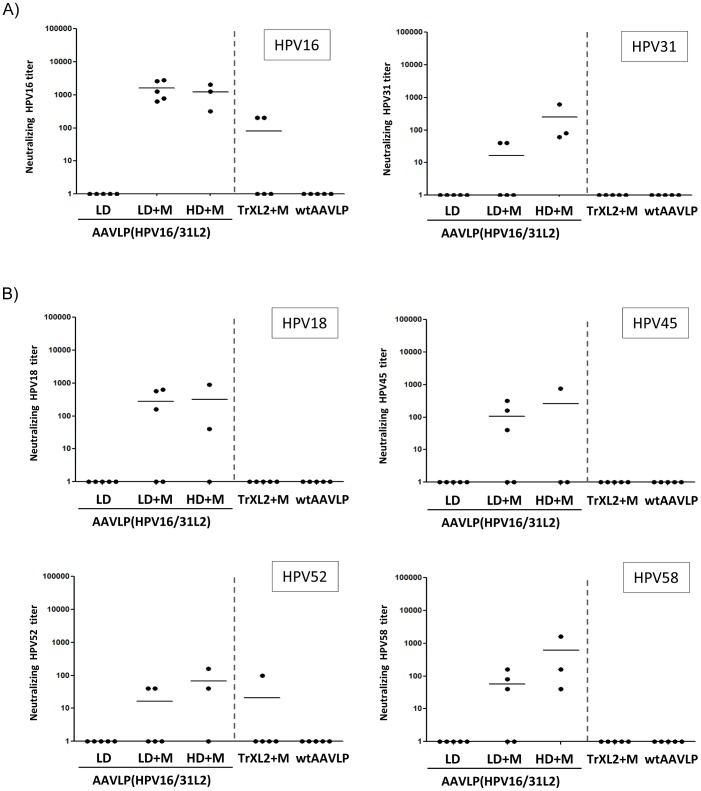
Detection of neutralizing and cross-neutralizing antibodies after vaccination with AAVLP(HPV16/31L2) in C57BL/6 mice. A) Neutralizing antibodies in sera of immunized animals were analyzed by HPV16 and HPV31 pseudovirion infection assay. B) Cross-neutralizing antibodies against HPV18, HPV45, HPV52 and HPV58 pseudovirions were detected by pseudovirion infection assay. Data of individual mice are shown. Data are expressed as the reciprocal neutralization titer obtained by using a cut off of 50% neutralization activity by incubation with serial dilutions from 1∶40 to 1∶30,720 of the sera. Mean titers of each group are shown by horizontal bars.

Neutralization was determined by inhibition of gene transduction by pseudovirions (PsV) containing the gene for gaussia luciferase (gLuc). Transduction was measured by gLuc expression which is taken as a surrogate of infection. The sera were tested in serial dilutions from 1∶40 to 1∶30,720, and a 50% reduction of gLuc activity was defined as positive neutralization. As shown in figure 3A, the enhancing effect of the adjuvant was also evident from the neutralizing activity against HPV16 and HPV31, but the HPV31-specific neutralization was weaker. Future HPV vaccines should induce immunity against other high-risk HPV types. We therefore analysed the ability of our particles to induce cross-neutralizing antibodies against several PsV (HPV18, HPV45, HPV52 and HPV58). As shown in figure 3B a similar pattern of reactivity was observed when the sera were tested for cross-neutralizing antibodies against HPV18, HPV45, HPV52 and HPV58. Animals vaccinated with TrXL2+M did not develop cross-neutralizing antibodies. We conclude from these results that neutralizing activity against HPV16 induced by the particles is higher than the one to HPV31. Cross-neutralization can only be achieved with adjuvanted AAVLPs. Reactivity of sera from mice immunized with AAVLP(HPV16/31L2) particles was higher than TrXL2 in C57BL/6 mice.

### Immunogenicity of AAVLP(HPV16/31L2) Particles in Balb/c Mice

Cross-neutralizing activity of AAVLP(HPV16/31L2) was more closely analyzed in Balb/c mice, to this end animals were immunized with low dose of AAVLP plus montanide ISA51 two (LD+M 2x) or three times (LD+M 3x). In order to test the potency of another adjuvant a group of mice were vaccinated three times i.m. with low dose of AAVLP together with monophosphoryl Lipid A (MPL) (LD+MPL). Animals vaccinated with TrXL2 plus montanide ISA51 (TrXL2+ M) or with one of the two licensed HPV vaccines, Cervarix™ and Gardasil™ (1/10 of the dose recommended for humans) were used as controls.

**Figure 4:**
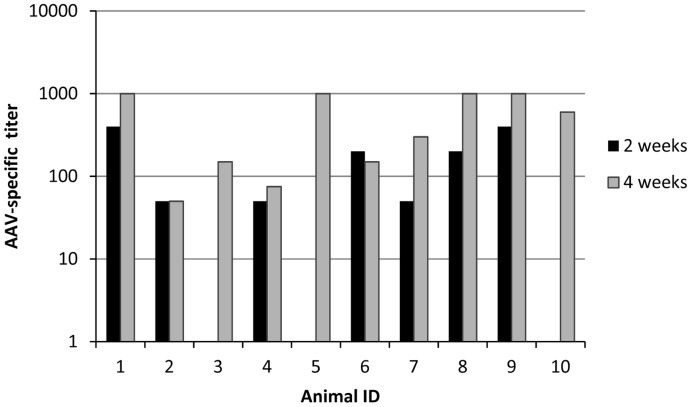
Detection of AAV-specific antibodies in Balb/c mice. Ten Balb/c mice were immunized twice intranasally with AAV2 (1E+11 capsids). Two (black bars) and four weeks (grey bars) after the first immunization sera was tested for detection of AAV-specific capsid antibodies using an AAV-based ELISA. Data are expressed as reciprocal titers of individual animal.

**Figure 5:**
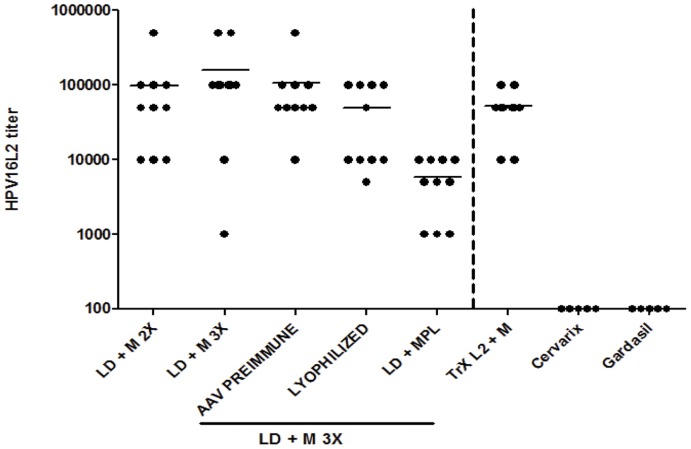
Detection of HPV16 L2-specific antibodies in Balb/c mice vaccinated with AAVLP(HPV16/31L2). Mice were immunized with low dose of AAVLP(HPV16/31L2) particles either twice (LD+M 2x) or three times (LD+M 3x) i.m. together with montanide ISA51 as an adjuvant. A group of animals were vaccinated three times intramuscularly (i.m.) with low dose of AAVLP(HPV16/31L2) particles together with monophosphoryl Lipid A (MPL) as an adjuvant (LD+MPL). In addition, one group of mice was immunized with three low doses of the lyophilized AAVLP(HPV16/31L2) particles in combination with montanide ISA51 (Lyophilized). Four weeks after vaccination with AAV2 a group of animals were i.m. vaccinated with AAVLP(HPV16/31L2) three times with montanide ISA51 (AAV preimmune). Animals vaccinated 3 times subcutaneously (s.c.) with thioredoxin-L220–38 (50 µg) plus montanide ISA51 (TrXL2+ M) and a group of animals immunized with Cervarix™ and Gardasil™ were used as controls. Six weeks after the first immunization HPV16 L2-specific antibodies were detected by GST-L2 ELISA. Data of individual mice are shown. Mean titers of each group are shown by horizontal bars.

A potential challenge for using AAV2-based vaccines in humans is the naturally acquired immune responses in approximately 80% of the human population ([24], [25], [15]), as the presence of antibodies against the AAV capsid may prevent an efficient vaccination. Therefore we immunized a group of animals with AAV2 (1E+11 particles/animal) prior to vaccination with AAVLP. Two and four weeks after pre-immunization, antibodies reacting with AAV2 capsids were detected by ELISA (titers between 80 and 1000) (figure 4). At week 4 animals were vaccinated with LD+M 3x. Generation of HPV16 L2 specific antibodies was measured by a GST-L2 ELISA two weeks after the last immunization. As shown in figure 5, mice immunized with two or three doses of AAVLPs as well as with TrXL2+M developed comparable high titers of L2-specific antibodies (>100,000). Interestingly, also after pre-immunization with AAV similar titer levels were measured. The requirements for vaccines that are stable at ambient temperature imply the development of freeze-dried formulations may be beneficial. Lyophilized and rehydrated AAVLP(HPV16/31L2) particles were shown to be only slightly less immunogenic compared to the unprocessed particles. Animals vaccinated in the presence of MPL developed around one log lower HPV16 L2-specific antibody titers (<10,000) than mice which received montanide ISA51 adjuvanted particles.

**Figure 6:**
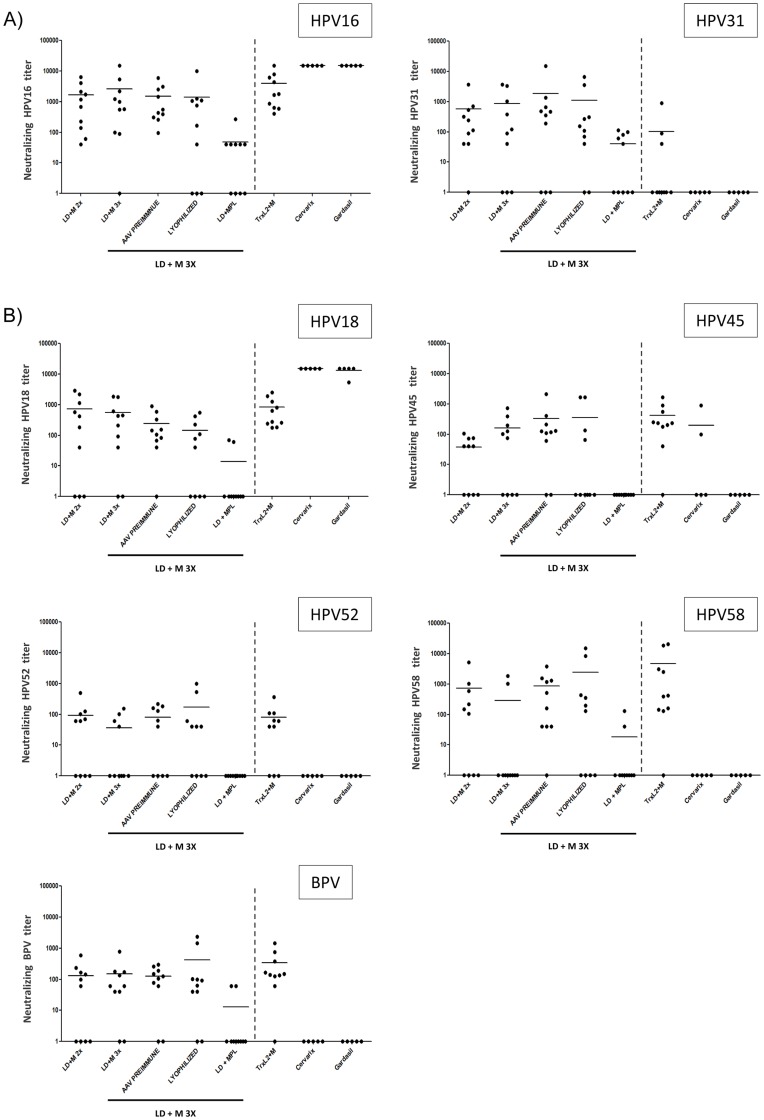
Detection of neutralizing and cross-neutralizing antibodies after vaccination of Balb/c mice with AAVLP(HPV16/31L2). A) Neutralizing antibodies in sera of immunized Balb/c animals were analyzed by HPV16 and HPV31 pseudovirion infection assay. B) Cross-neutralizing antibodies against HPV18, HPV45, HPV52, HPV58 and BPV pseudovirions were detected by pseudovirion infection assay. Data of individual mice are shown. Data are expressed as the reciprocal neutralization titer obtained by using a cut off of 50% neutralization activity by incubation with serial dilutions of the sera. Mean titers of each group are shown by horizontal bars.

Neutralizing activity of the different groups against HPV16 and HPV31 PsV followed a similar pattern as the titers measured by ELISA with responses against HPV31 being consistently lower than against HPV16 (figure 6A). Sera of all mice vaccinated with TrXL2+M, Cervarix™ and Gardasil™ had neutralizing activity against HPV16 PsV with mean titers of >10000 but not HPV31 PsV. Cross-neutralizing antibodies against HPV18, HPV45, HPV52, HPV58 and BPV PsV were detected, again in a similar ratio between the groups with neutralizing activity being highest against HPV18, HPV45 and HPV58 (figure 6B). Most of the animals immunized with TrXL2+M developed HPV45, HPV52, HPV58 and BPV cross-neutralizing antibodies. Low level of cross-reactivity against HPV45 was obtained with 2/5 animals that had received Cervarix™.

In conclusion, Balb/c and C57BL/6 mice responded with similar HPV16- specific titers to immunization with AAVLP(HPV16/31L2) particles, but the cross-neutralizing titers were higher in Balb/c mice. Remarkably, pre-existing AAV antibodies did not prevent the induction of humoral responses against HPV. Lyophilized particles retained immunogenicity after reconstitution, albeit at somewhat lower level. In both mouse strains immune responses against HPV16 induced by AAVLP is higher than against HPV31, suggesting that the HPV31 L2-epitope inserted at position 453 is less immunogenic than the HPV16 L2-epitope inserted at aa 587.

### Immunogenicity of AAVLP(HPV16/31L2) Particles in Rabbits

**Figure 7:**
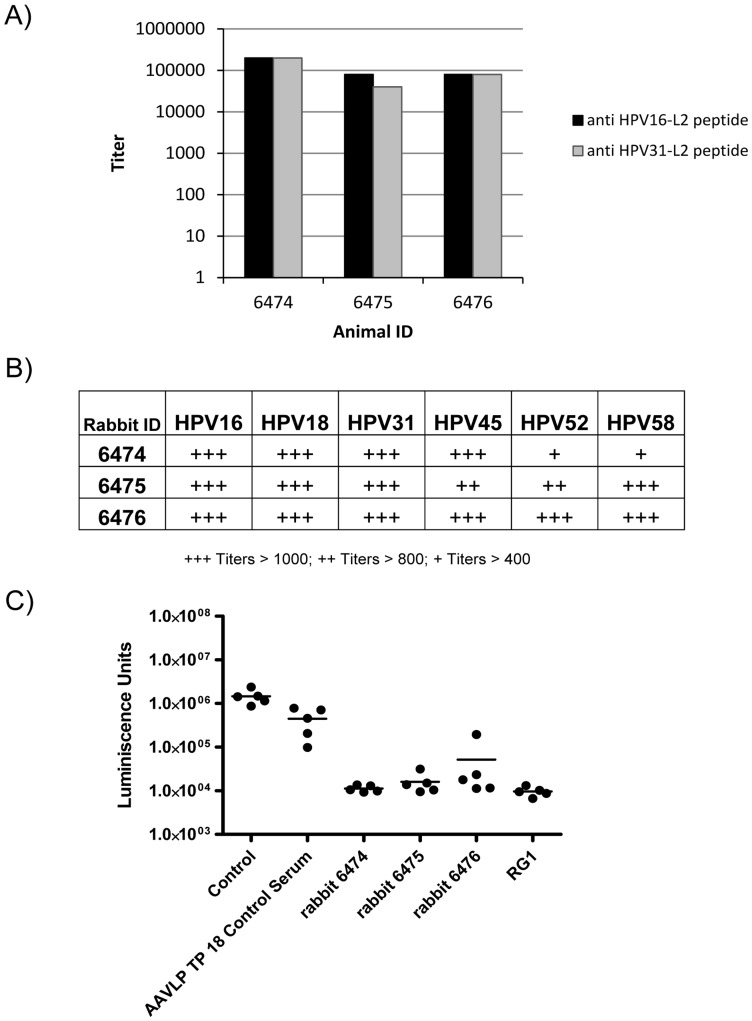
Immunoresponse of AAVLP(HPV16/31L2) in rabbits. Three rabbits were immunized i.m. four times with AAVLP(HPV16/31L2) particles (2E+12 capsids equivalent to 13.2 µg) together with montanide ISA720 as an adjuvant. A) Ten weeks after the first immunization sera was analyzed by an L2 peptide-based ELISA to detect HPV16 and HPV31 L2-specific antibodies. B) The presence of neutralizing and cross-neutralizing antibodies against six different HPV types (HPV16, 18, 31, 45, 52, 58) was measured by pseudovirion-based neutralization assay (PBNA). C) Naïve Balb/c mice were injected i.p. with 100 µl buffer, 20 µg RG-1 purified monoclonal antibody or rabbit antiserum to AAVLP TP18 or serum from three different rabbits vaccinated with AAVLP(HPV16/31L2). One day later, the mice were vaginally challenged with HPV16 pseudovirus carrying a luciferase reporter. At three days post challenge, infection was assessed by imaging luciferase activity.

In addition, three ZIKA hybrid rabbits were immunized four times with AAVLP(HPV16/31L2) particles (2E+12 capsids equivalent to 13.2 µg) adjuvanted with montanide ISA720. When analyzed two weeks after the last immunization, all animals developed HPV16/31 L2-specific antibodies with titers around 100,000 (figure 7A). The antibodies neutralized PsV of HPV16 and HPV31 at dilutions higher than 1∶1000 (figure 7B). Cross-neutralizing titer against HPV18 was similar, whereas cross-neutralization of HPV45, HPV52 and HPV58 PsV was lower but still significant. These data support the high and broad reacting immune response induced by AAVLP(HPV16/31L2) particles also in this animal model.

To test whether the rabbit sera raised against AAVLP(HPV16/31L2) confer protection against HPV16 infection, in an *in vivo* model, rabbit sera were administered intraperitoneally to naïve mice. Rabbit sera against AAVLP with an epitope of the cholesteryl ester transfer protein (AAVLP TP18) were used to detect the unspecific effect of AAVLP induced antibodies. Vaginal infection of mice is detected three days after challenge as luminescence signal after injection of the challenged mice with luciferin. The passive transfer of AAVLP(HPV16/31L2) sera protected mice from vaginal challenge with HPV16 PsV, whereas the AAVLP TP18 control serum failed to protect the mice (figure 7C). Protection by AAVLP(HPV16/31L2) sera was similar to RG-1, a neutralizing monoclonal antibody recognizing the L2 17–36 motif.

## Discussion

The first steps in papillomavirus infection involve L1 dependent binding of the viral capsid to heparan sulfate proteoglycan [26] and lamin 5 in the extracellular matrix [27] which induces a conformational change leading to the exposure of L2, furin dependent proteolytic processing and attachment to keratinocytes [28], [29]. Intervention with this infection pathway by prophylactic vaccination has two possible targets, the L1 or the L2 protein of the HPV capsid. While L1 antibodies are able to prevent both steps – attachment to basal membrane and to keratinocytes – L2 antibodies can interfere only with binding to the keratinocytes. L1 antibodies can be induced to high neutralization titers, especially when generated by HPV-VLPs, however, they have the drawback of HPV-type restriction. Furthermore, L1 VLPs are expensive to produce, especially for highly multivalent vaccines, and distribution of such vaccines requires a cold chain which limits their adoption by developing countries. Thus, although two approved L1 HPV-VLP vaccines – Gardasil™ and Cervarix™ – are highly protective against the main high-risk HPV infections, they lack cross-protection against other, more rare high-risk HPV types, they are not heat stable and are expensive to produce. L2 vaccines on the other hand, are able to expand the cross-protection profile due to extensive homology of the N terminus of L2 [30]. In particular, oligopeptides of the L2 proteins are sufficient to induce a broadly cross-neutralizing immune response [31]. However, neutralizing titers generated by L2 peptides are relatively low compared to those produced by L1VLPs [32].

Several attempts have been made to improve the immunogenicity of L2. These include insertion of L2-neutralizing epitopes on the surface of HPV VLPs [9], [33], construction of tandem repeats of one peptide on bacterial thioredoxin [3] or concatenated L2 peptides from different papillomavirus types [11]. We have chosen AAVLPs as a platform for presentation of a cross-neutralizing L2 epitope (aa 17–36). AAV capsids tolerate insertion of peptides at different positions of the capsid and can be produced in large quantities from only one capsid protein making it a simple, non-infectious, empty capsid vaccine molecule [13], [18], [19], [20]. In this first exploration of AAVLPs as a vaccine for generating a humoral immune response, the anti-peptide titers obtained were remarkably high (in the range of 10^4^ to 10^5^) after two immunizations with only 0.6 µg VLPs per dose. A third immunization only slightly increased the anti-L2 peptide titers. Nevertheless, an adjuvant seems to be required for AAVLP vaccination, because the recombinant AAV capsid alone elicits only a weak or no innate immune response. So far, only one alternative adjuvant (MPL) has been tested which was not as effective as montanide. Other options have still to be explored [34], [35]. Pre-existence of anti AAV antibodies which are highly prevalent in the human populations, rather boosts than prevents the generation of the L2 specific immune response. This could be explained by improved, anti-AAV antibody Fc-mediated uptake of AAVLPs by professional antibody presenting cells. A particular advantage of AAVLPs is their stability which allows lyophilization and rehydration [36]. Thus, lyophilization of AAVLPs likely permits storage and delivery of AAVLPs under harsh conditions and in principle circumvents the need of a cold chain for vaccine distribution. This may be an important cost factor in developing countries and should be further explored.

The AAVLPs of this study can be produced by transient transfection of one or two plasmids expressing the capsid protein VP3 and the assembly activating protein AAP [13]. Under these conditions production of 10^12^ to 10^13^ capsids/ml can be achieved. Purification of the particles follows standard chromatography procedures and does not constitute a bottle-neck for AAVLP manufacturing. Co-expression of VP3 and AAP using baculovirus based vectors in insect cells provides one possibility of scale-up for clinical applications ([37], our own results). Methods for less costly AAVLP production, e.g. using bacteria or yeast, have still to be explored.

Immunization with AAVLPs containing the insertion of two different L2 peptide sequences at two separate positions of the same capsid provided a broadly cross-neutralizing antiserum, as tested in a pseudovirion (PsV) infection assay. This was observed in C57BL/6 mice, Balb/c mice and in rabbits. The HPV16-L2 peptide inserted at 587 provided nearly 100% protection against HPV16 PsV infection with rather high neutralizing antibody titers. This was not the case for the HPV31-L2 peptide inserted at aa position 453 and tested in the HPV31 PsV-based neutralization assay. The result is in agreement with the study of Jagu et al. that showed that a multitype L2 fusion protein which included a peptide of HPV31 induced robust antibody titers against various HPV types, but had relatively low titers against HPV31 [11]. However, also the position of peptide insertion in the AAV capsid may play a role. Peptides at position 453 are more exposed on the AAV capsid surface and have a larger interpeptide distance at the threefold symmetry axis of AAV2 than peptides inserted at aa 587. Immunization with the same peptides inserted at the reversed positions should show whether the inherent immunogenicity of the two peptides or the localization on the capsid surface is responsible for the difference in the immune reaction. Such an analysis might provide valuable information for the optimal spatial arrangement of antigenic epitopes on viral scaffolds for vaccination.

Because vaccination at low dose applies only 0.6 µg of AAVLPs/dose, a number of combinations of different AAVLPs can be tested for optimization of the vaccine. This includes also combinations of different AAVLPs with insertions of different HPV-L2 peptides at one position or combined at two positions. Another option would be the insertion of L2 peptides into other immunogenic sites of the AAV capsid [38]. Furthermore, the combination of peptide insertions with the expression of antigens by gene transfer may provide a basis for a combined prophylactic and therapeutic vaccine.

Cross-neutralizing vaccines against HPV types may not only be prophylactic for cervical cancer and genital warts, but may also have an impact on the prevention of a subset of head and neck or skin cancers for which a causal role of certain HPV types has been described. In general, this study describes the potential of AAVLPs as a vaccination option for prevention of infection associated diseases upon insertion of the appropriate linear protective epitope.

## Materials and Methods

### Ethics Statement

All animal procedures were performed according to approved protocols and in strict accordance with institutional and national guidelines; Federal law and the standard ethical guidelines (NIH, 1985; European Communities Directives, 1986 86/609/EEC) and approved by local government authorities (Regierungspräsidium Karlsruhe, Germany), guide for the Care and Use of Laboratory Animals of the National Institutes of Health, with the prior approval of the Animal Care and Use Committee of Johns Hopkins University. All immunizations were performed under ketamine anesthesia as describe below, and all efforts were made to minimize suffering.

### Cell Lines and Culture Conditions

Human embryonic kidney (HEK) 293T cells [39], and 293TT cells [40] were maintained in Dulbecco modified Eagle medium (DMEM) supplemented with 10% heat-inactivated fetal-calf serum, 100 U of penicillin/ml, and 100 µg of streptomycin/ml at 37°C in 5% CO_2_. The HeLaT cell line was generated by transfecting a linearized expression plasmid with T-Ag under the CMV promoter, followed by selection with hygromycin. Several clones were screened with pseudovirions for best luciferase transduction.

### Animals

C57BL/6 (*H-2b*) and Balb/c female mice at 6–8 weeks of age were purchased from Charles River Wiga (Sulzfeld, Germany) and kept in an isolator at the animal facilities of the DKFZ. ZIKA hybrid rabbits were purchased from Dr. Zimmermann GbR (Abtsgmünd- Untergröningen, Germany) and housed at the company BioGenes (Berlin, Germany). For HPV challenging female Balb/c (6–8 weeks old) were purchased from the National Cancer Institute (Frederick, MD, USA) and kept in the animal facility of the Johns Hopkins School of Medicine (Baltimore, MD, USA).

### Cloning of AAVLP(HPV16/31L2)

AAVLP particles were generated from a plasmid containing the overlapping AAV2 VP2 and VP3 coding sequence cloned into the *Xho*I and *Not*I site of the pCI plasmid (Promega, Madison, WI). The start codon of VP2 was destroyed by introducing a point-mutation using the Quick Change Site-Directed Mutagenesis kit (Agilent Technologies, La Jolla, CA). A *Fse*I site was introduced without changing the VP3 protein sequence at position 520 (amino acid number relative to the VP1 protein of AAV2) to generate the plasmid pCIVP2mut. In order to introduce peptides into VP3 the plasmid pCIVP2mut was modified. The plasmid pCIVP2mut-I587 was generated by introduction of *Not*I and *Bsp*EI sites at position 587. A second plasmid, pCIVP2mut-I453, was generated by introduction of *No*tI and *Bsp*EI sites at position 453. The nucleotide sequence of L2 residues 17 to 36 of HPV16 was cloned into the *NotI/Bsp*EI digested pCIVP2mut-I587 and the nucleotide sequence of L2 residues 17 to 36 of HPV31 into the *NotI/Bsp*EI digested pCIVP2mut-I453. Subsequently a fragment containing the HPV31 L2 sequence was generated by digestion of pCIVP2mut-I453 with *Bsi*WI/*Fse*I. The fragment was subcloned into the *Bsi*WI/*Fse*I digested pCIVP2mut-I587 and finally arginines at position 585 and 587 were substituted by alanine residues to generate the plasmid for the AAVLP(HPV16/31L2) production.

### Production and Purification of AAVLP(HPV16/31L2)

293T cells were transfected with AAVLP(HPV16/31L2) plasmid DNA (36 µg per 15 cm dish). Transfected cells were harvested after 3 days and lysed by three freeze-thaw cycles. The medium was cleared by filtration and adjusted to pH to 6.0 in HEPES buffer. Particles were further purified through chromatography. Briefly, the diluted cleared lysate containing the AAVLP particles was loaded onto Fractogel EMD SO_3_ column and after washing with HEPES buffer were eluted with 0.6 M NaCl. After buffer exchange the particles were processed through CaptoQ column (GE Healthcare). The particles in the flow-through, were concentrated and separated through a Superdex 200 column (GE Healthcare). Fractions containing AAVLP(HPV16/31L2) particles (analyzed by SDS-PAGE) were pooled and sterile filtrated. The particle titer was determined using the AAV2 titration ELISA (Progen, Heidelberg, Germany). Endotoxin concentration was measured using the turbimetric kinetic LAL test (BSL Bioservice, Planegg, Germany).

### Western Blotting and Dot Blot Analysis

Expression of the AAVLP(HPV16/31L2) VP3 proteins was verified by Western blotting using the monoclonal antibody B1 (diluted 1∶200, Progen). The blotted membrane was incubated with 5% skim milk in 1 x PBS/0.05% Tween-20 for 1 h at RT followed by incubation of the membrane with antibody B1 for 1 hour at RT. After washing bound antibodies were detected with 1∶2,500 diluted HRP-labeled anti-mouse IgG (DAKO, Glostrup, Denmark). The expression of the AAVLP HPV16 and HPV31 L2 inserted peptides was analyzed by native dot blotting. Two µg (3E+11 particles) of the AAVLP(HPV16/31L2) particles, 2 µg of AAVLP particles without inserts or 10 µg of the HPV16 and 31 L2 peptides QLYKTCKQAGTCPPDIIPKV and QLYQTCKAAGTCPSDVIPKI were dotted on a nitrocellulose membrane that was incubated with 1∶1,000 diluted rabbit sera for 1 hour at RT. Bound antibodies were detected with 1∶2,500 diluted HRP-labeled anti-rabbit IgG (DAKO, Glostrup, Denmark).

### Lyophilization

Lyophilization of particles in 600 mM mannitol dissolved in Hepes buffer pH 6.0 with 200 mM NaCl was carried out using Epsilon 2-12D freeze-drier (Martin Christ Freeze Dryers GmbH, Osterode, Germany) stored at ≤20°C until use.

### TEM

Transmission electron microscopy (TEM) of the purified AAVLP(HPV16/31L2) particles was performed after fixation 2.5% glutardialdehyde and stained with 2% uranium acetate and 0.01% glucose (performed by Prof. Dr. Wanner at the LMU Munich, Germany).

### AAV2 Empty Capsid Production

Particles were produced as described previously in [36]. Briefly, 293T cells were infected with a recombinant adenovirus expressing VP1, VP2 and VP3 of AAV2. Cells were harvested and fractionated on a sucrose cushion by under laying the supernatant with 450 µl of 30% sucrose (in Tris-EDTA) followed by 450 µl of 50% sucrose in Tris-EDTA. The gradients were centrifuged at 273,000×*g* for 2.5 h at 4°C by using an SW60 rotor (Beckman, Germany). The pellet was resuspended in TBSM buffer and purified on a second sucrose cushion. The resulting pellet was resuspended in an appropriate volume of TBSM buffer and then analyzed by electron microscopy.

### Immunization of Animals

Female mice were kept under deep ketamine 10%/rompun 2% (Bayer) anesthesia by intraperitoneal injection. Low doses (1E+11 particles/dose) or High doses (5E+12 particles/dose) of AAVLP(HPV16/31L2) were delivered intramuscularly into the tibia anterior muscle of the right leg with or without adjuvant. Two weeks later a boost was administrated in the same way. In case of TrX-L2 immunization, 50 µg of filter-sterilized TrX-L2(20–38)_3_ [3] mixed with adjuvant was applied subcutaneously. Two and four weeks later, two boosts were administrated in the same way. Samples of blood were collected 45 days after the first immunization. All samples were stored at −20°C. Female rabbits were immunized intramuscularly with 2E+12 capsids/dose in a 1∶2 mixture of montanide ISA720, followed by boosts two weeks, five weeks and eight weeks later (BioGenes). Sera were collected 14 days after the last boost and stored at −20°C. For control purposes one female rabbit was immunized with AAVLPs containing an epitope of the cholesteryl ester transfer protein (AAVLP TP18) [41].

### Adjuvants

Montanide ISA51 and montanide ISA720 were obtained from Seppic (Paris, France). A mixture of 1∶1 (ISA51) or 1∶2 (ISA720) with the antigen was applied per dose. Five micrograms of monophosphoryl lipid A derived from *Salmonella enterica* (MPL) in 2% squalene oil-in-water emulsion (Sigma Adjuvant System, Sigma, St. Louis, MA) were applied per dose.

### Measurement of Antibody Responses

The presence of HPV16 L2-specific IgG antibodies in sera of immunized mice was determined by GST L2-ELISA as described [3]. To detect anti-AAV antibodies sera (in 2-fold dilutions starting from 1∶50 to 1∶500,000) were added into AAV2 pre-coated plates (10^10^ particles/well). HRP-coupled antimouse IgG was used as secondary antibody (dil 1∶5000, Southern Biotechnology, Birmingham, MA). TMB (Sigma) substrate solution was used as substrate OD was measured in an ELISA reader at 450 nm. Nonspecific binding was determined by using the same dilutions on plates coated with PBS only. The presence of HPV16 and HPV31 L2-specific IgG antibodies in sera of immunized rabbits was determined by L2 peptide-ELISA. 96-well plates were coated with 1 µg/well of the peptides QLYKTCKQAGTCPPDIIPKV or QLYQTCKAAGTCPSDVIPKI in PBS overnight at 4°C and blocked with 5% skim milk in PBS-T for 1 h at 37°C. The peptides were incubated with serial dilutions of the sera (3-fold dilutions starting from 1∶1,000 to 1∶2,187,000) in PBS-T with 1% skim milk and 1% BSA for 1 h at 37°C. Rabbit pre- immune sera were diluted 1∶1,000. Bound rabbit IgGs were detected after incubation with a 1∶2,500 diluted HRP-labeled anti-rabbit IgG (DAKO) antibody following the same conditions as above.

### Pseudovirion-based Neutralization Assay

Neutralizing antibodies in sera of immunized animals were determined as described previously [4] but with pseudovirions (PSV) expressing gaussia luciferase (gLuc) as reporter. Briefly, PsV were prepared by transfecting 293TT cells with a plasmid encoding for the humanized HPV16 L1 and L2 genes, together with a plasmid containing the gene for gLuc under the control of CMV promoter. For PsV extraction, 293TT cells were harvested and washed once with 1 ml of Dulbecco’s Phosphate Buffered Saline (DPBS; Gibco). The cell pellet was resuspended in 150 µl of lysis buffer (140 µl DBPS, 9 µl 10% Brij58 (w/v) (Sigma) and 1 µl RNAse A/T cocktail (Fermentas)). The PsV-containing supernatant was set aside and the cell pellet was resuspended anew in 300 µl DPBS/0.8 M NaCl. Samples were cleared by centrifugation at 10,000 rpm and 4°C for 10 minutes. The supernatants were combined, 2 µl benzonase were added and the samples were incubated for 1 hour at 37°C. The PsV were Optiprep purified and used for infection of HeLaT cells.

Sera were transferred to 384 well polypropylene V-bottom plates (Greiner) and serially diluted ten times in 2-fold increments from 1/3 to 1/1536. Each 2 µl of the serially diluted sera were transferred to 21 white 384 well cell culture plates (PerkinElmer) with an EP3 384-head pipetting robot (Perkin Elmer), the plates immediately sealed with a polypropylene cover foil and stored at −20°C.

### In Vivo Pseudovirus Delivery and Infection

HPV16 pseudovirions with encapsulated luciferase reporter were generated by cotransfection of 293TT cells with plasmids encoding codon-modified L1 and L2 and a firefly luciferase reporter plasmid, as described previously [40]. Female Balb/c mice were treated with 3 mg of Depo-provera (Pfizer) 4 days prior to viral challenge. One day prior to challenge, mice were injected i.p. with buffer, undiluted rabbit antisera or 20 µg of purified RG-1 monoclonal antibody. The mice were anesthetized and challenged with pseudovirus as previously described [42], but with modifications. Briefly, a HPV16 PsV inoculum of 20 µl mixed with 20 µl of a 3% CMC preparation, based on L1 content, was used. The inoculum was delivered using an M20 positive-displacement pipette in two doses; 20 µl before and 20 µl after insertion in the vagina of a Cytobrush that was turned clockwise and counterclockwise 10 times. After the challenge, standard dissecting forceps were used to occlude the vaginal introitus to achieve maximal retention of the material while the mice recover from anesthesia. Three days after pseudovirion challenge, the mice were again anesthetized and 20 µl of luciferin (7.8 mg/ml) was deposited in the vaginal vault. Luciferase signals were acquired for 10 min with a Xenogen IVIS 100 imager, and analysis was performed with Living Image 2.0 software (Caliper Life Sciences). An identical region of interest (ROI) was drawn around the luciferase signal emitted from each mouse, and the average radiance within the ROI was determined.
